# Differential Characterization of Two Kinds of Stem Cells Isolated from Rabbit Nucleus Pulposus and Annulus Fibrosus

**DOI:** 10.1155/2016/8283257

**Published:** 2016-09-15

**Authors:** Chenglin Sang, Xuecheng Cao, Fangjing Chen, Xuekang Yang, Yongxian Zhang

**Affiliations:** ^1^Department of Orthopedics, Second Military Medical University's Jinan Clinical Medicine College, 25 Shifan Road, Jinan, Shandong 250031, China; ^2^Department of Orthopedics, General Hospital of Jinan Military Command, 25 Shifan Road, Jinan, Shandong 250031, China; ^3^Department of Burns and Cutaneous Surgery, Xijing Hospital, Fourth Military Medical University, No. 127 Changle West Road, Xi'an, Shaanxi 710032, China

## Abstract

*Objective*. Nucleus pulposus (NP) and annulus fibrosus (AF) are two main components of intervertebral disc (IVD). We aimed to figure out whether NP and AF also contain stem cells and whether these stem cells share common properties with chondrocytes and/or fibroblasts in their phenotypes or whether they are completely different types of cells with different characteristics.* Design*. The disk cells were isolated from AF and NP tissues of the same lumbar spine of the rabbits. The properties of these disk cells were characterized by their morphology, population doubling time (PDT), stem cell marker expression, and multidifferentiation potential using tissue culture techniques, immunocytochemistry, and RT-PCR.* Results*. Both disk cells formed colonies in culture and expressed stem cell markers, nucleostemin, Oct-4, SSEA-4, and Stro-1, at early passages. However, after 5 passages, AFSCs became elongated and NPSCs appeared senescent.* Conclusion*. This study indicated that IVD contains stem cells and the characteristics of AFSCs and NPSCs are intrinsically different. The findings of this study may provide basic scientific data for understanding the properties of IVD cells and the mechanisms of lower back pain.

## 1. Introduction

Low back pain (LBP) is one of the most common health problems in humans and affects about 80% of the population at some time during their lifetime [1, 2]. Intervertebral disc (IVD) plays an important role in the integrity of vertebral column function and intervertebral disc degeneration (IVDD) is the most frequent cause of low back pain [3]. The cost of chronic low back pain exceeds the combined costs of stroke, respiratory infection, diabetes, coronary artery disease, and rheumatoid disease [4].

To date, no curative treatment is available for IVDD and the management includes bed rest, physiotherapy, and analgesic pain relief [5]. Traditional treatments, including surgical and pharmacological therapy, target pain relief but fail to address the underlying pathology due to the lack of understanding the properties of IVD cells [6].

The IVD contains three components: nucleus pulposus (NP), annulus fibrosus (AF), and the cartilaginous end plate (EP). NP cells are chondrocyte-like cells situated within a collagen type II and proteoglycan-enriched matrix that absorbs the spinal load. AF cells are fibroblast-like cells that are embedded in a collagen type 1-enriched expansion-resistant matrix. EP is composed of hyaline cartilage that connects the IVD to the bone of the neighboring vertebra [7]. Structural or compositional alterations in any of the three components alter the disc's ability to withstand load applied to the spine. The exact mechanisms that induce the IVDD-associated pain are not well understood, but it is assumed that its development may be secondary to inflammation and injury or age-related. In all cases, changes occurring in disc structure are identical and are initially noted in the EP, followed by alterations in the NP composition and finally in the AF.

Currently, therapeutic interventions for the treatment of LBP and IVD degeneration are largely conservative. Novel regenerative techniques have been considered, including the use of gene therapy and the injection of antagonists against the upregulated proinflammatory cytokines into the degenerate discs [8, 9]. However, side effects associated with the use of viral vectors and the need for frequent administration means such techniques are not optimal.

Thus, the use of stem cells has received increased interest regarding IVD regeneration. In recent years, a tissue engineering approach has been sought to improve the structure and function of degenerated IVD using stem cell therapy. A common source of stem cells used to repair injured tissues is bone marrow mesenchymal stem cells (BMSCs) [10]. BMSCs are multipotent cells that can differentiate into several cell types, including chondrocytes and osteoblasts. BMSC therapy, therefore, offers a promising treatment option for damaged cartilage and bone. BMSCs have also been used in the repair of degenerated IVD, but, in many cases, the ectopic bone was formed within IVDs [11].

Besides BMSCs, adult cells, such as dermal fibroblasts and autologous NP and AF cells, have also been used to treat degenerated IVDs, meeting with varying degrees of success [12]. Therefore, the development of new effective cell therapies for restoration of normal IVD structure and function is highly desirable, but progress has been hindered by a lack of characterization of IVD cells.

Recently, remarkable progress has been made with the identification of stem/progenitor cells in various tissues, such as tendons [4] and fatty tissues [13]. Adult stem cells are characterized by their multidifferentiation potential; under some conditions, the adult stem cells can differentiate into adipocytes, chondrocytes, and osteocytes. As IVD contains predominantly AF cells and NP cells, these previous studies raise the questions of whether stem cells also exist in IVD tissue and share common properties with chondrocytes and/or fibroblasts including NP cells and AF cells in their phenotypes or whether they are completely different types of cells with different characteristics.

We hypothesized that NP and AF tissues contain stem cells. These stem cells differ from fibroblast-like chondrocytes in proliferative and differentiation potentials, cell marker expression, and morphology and are good candidates for degenerate disc regeneration. To test our hypothesis, we used young rabbits (8–12 weeks old) to isolate stem cells from NP and AF for characterizing their cellular properties. No studies to date have compared the properties of the stem cells isolated from NP and AF of rabbits, which are often used as an animal model for the study of IVD healing and biomechanics due to their relatively large size and low cost for* in vivo* experiments.

## 2. Materials and Methods

### 2.1. Isolation of Stem Cells from AF and NP Tissues

The NP and AF regions were obtained from lumbar spine of five New Zealand white rabbits (8–12 weeks old, 3.0 to 4.0 kg weight). The protocol for using rabbits has been approved by IACUC of the Second Military Medical University, China. All rabbits were injected with ketamine (40 mg/kg) and xylazine (3 mg/kg) for sedation and were then sacrificed using pentobarbital (120 mg/kg).

After the animals were sacrificed, the AF tissues were weighed and cut into small pieces (1 mm × 1 mm × 1 mm). The gelatinous NP tissues from the central part of the disc were weighed and transferred to a tube directly without cutting. The AF tissue samples (100 mg) were digested with 1 mL of 0.4% pronase in PBS at 37°C for 1 hour. Then the AF tissue samples were treated with 1 mL of 0.04% collagenase P in PBS overnight at 4°C. The NP tissue samples (100 mg) were treated with 1 mL of 0.2% of pronase in PBS at 37°C for 1 hour. The NP tissue samples were reacted with 1 mL of 0.02% collagenase P in PBS overnight at 4°C. Each resulting cell suspension was passed through a 70 *μ*m cell sieve and washed three times with PBS. The PBS was removed by centrifugation at 500 g for 5 min. The cell pellets were resuspended in culture medium consisting of F-12 medium (Lonza, Walkersville, MD) supplemented with 1% penicillin-streptomycin solution and 20% fetal bovine serum (FBS; Atlanta Biological, Lawrenceville, GA). The cell suspension was cultured either in a 96-well plate at a density of 1 cell/well or T75 flasks at a density of 5 × 10^5^/flask, respectively. The morphology of the cells was watched and recorded by a microscope every day. The colony formation of both cells cultured in T75 flasks was examined by methyl violet staining at P0 stage. The colony numbers formed in each T75 flask were counted manually. The cells were then collected from all colonies by adding trypsin into the flask and counted using a hemocytometer.

For stem cell purification, 50 *μ*L of 0.25% trypsin was added into the single colony-containing well of a 96-well plate. If two or more colonies were found in the same well, 10 *μ*L of 0.25% trypsin was locally applied onto one colony to detach and pick up this colony under microscopic visualization according to the published protocol [14]. The detached individual cell colonies were collected using a micropipette and transferred to individual wells of a 6-well plate for further culture. After the cell colonies were removed from both AF and NP culture plates, some cells were still left in each tissue culture plate. These cells had a spindle shape and were spread around without colony formation. We named these cells as disc fibroblasts (DFCs) and added regular growth medium (DMEM plus 10% FBS) into culture plate for further culture using the published protocol [14].

### 2.2. Proliferation Assay

The proliferation of disc stem cells (AFSCs and NPSCs) and disc fibroblasts (DFCs) between passage 1 and passage 2 was studied by population doubling time (PDT) using the following calculation method [14]:

(1) PDT=duration×log⁡2log⁡final  cell  number−log⁡initial  cell  number.

### 2.3. Stem Cell Marker Analysis by Immunocytochemical Staining

The characterizations of disc stem cells (AFSCs and NPSCs) and disc fibroblasts (DFCs) were examined by immunocytochemical staining. The AFSCs and NPSCs and DFCs at passage 1 were seeded in 12-well plate at a density of 3 × 10^4^ cells/well and cultured for 5 days. The cells were fixed with 4% paraformaldehyde in phosphate-buffered saline (PBS) for 30 min at room temperature. The fixed cells were directly reacted with the following antibodies with different dilution for 2 hours at room temperature, such as mouse anti-stage-specific embryonic antigen-4 (SSEA-4; 1 : 500, Cat. # 414000, Invitrogen, Carlsbad, CA), mouse anti-Stro-1 antibody (1 : 350, Invitrogen, Cat. # 398401, Carlsbad, CA), mouse anti-collagen type I (1 : 350, Abcam, Cat. # ab90395, Cambridge, MA), mouse anti-collagen type II (1 : 350, Abcam, Cat. # ab3092, Cambridge, MA), and mouse anti-collagen type III (1 : 350, Abcam, Cat. # ab7778, Cambridge, MA). For octamer-binding transcription factor-4 (Oct-4) and nucleostemin (NS) staining, the fixed cells were further treated with 0.1% triton X-100 for 30 min at room temperature and then washed once with PBS. The triton X-100 treated cells were incubated either with mouse anti-Oct-4 antibody (1 : 350, Cat. #MAB4401, Millipore, Temecula, CA) or with goat anti-nucleostemin antibody (1 : 500, Cat. # GT15050, Neuromics, Edina, MN) for 2 hours at room temperature. After washing the cells three times with PBS, cyanine 3- (Cy3-) conjugated goat anti-mouse immunoglobulin G (IgG) secondary antibody (1 : 500, Cat. # A10521, Invitrogen, Carlsbad, CA) was used for Oct-4, SSEA-4, Stro-1, collagen type I, collagen II, and collagen III determination. In addition, nucleostemin was examined by Cy3 conjugated donkey anti-goat IgG antibody (1 : 500, Cat. # AP180C, Millipore, Temecula, CA). Finally, Hoechst 33342 (1 *μ*g/mL, Cat. # 33270, Sigma, St Louis) was used for total cell counterstaining in each well.

### 2.4. Multidifferentiation Potential of AFSCs and NPSCs

We also tested the multidifferentiation potential of AFSCs and NPSCs* in vitro *according to the published protocol [14]. AFSCs and NPSCs (2.4 × 10^5^ cells) at passage 1 were seeded in each well of a 6-well plate and cultured in low glucose-containing Dulbecco's modified Eagle's medium (DMEM) with 10% heat-inactivated FBS, 100 U/mL penicillin, and 100 *μ*g/mL streptomycin (basic growth medium) and cultured for two days. From the third day, the cells were cultured with either adipogenesis or osteogenesis or chondrogenesis differentiation media for 21 days. The media were changed every three days. The adipogenic differentiation medium was made by adding 1 *μ*M dexamethasone, 100 *μ*M indomethacin, 0.5 mM isobutylmethylxanthine (IBMX), and 10 *μ*g/mL insulin into the basic growth medium. The osteogenic differentiation medium consisted of basic growth medium plus 10 mM glycerol 2-phosphate, 200 *μ*M ascorbic 2-phosphate, and 100 nM dexamethasone. The chondrogenic induction medium was prepared by adding 40 *μ*g/mL proline, 10 ng/mL TGF-*β*3, 50 *μ*g/mL ascorbic 2-phosphate, 39 ng/mL dexamethasone, 100 *μ*g/mL sodium pyruvate, and 50 mg/mL insulin-transferrin-selenious acid mix (ITS; BD Biosciences, Bedford, MA) into basic growth medium. AFSCs and NPSCs cultured in basic growth medium were used as control cells, respectively. The differentiated cells were tested by Oil Red O for adipocytes, Alizarin Red S for osteocytes, and Safranin O for chondrocytes, respectively, according to previous studies [14–16]. The stained cells were examined on an inverted microscope, and the lipid droplets of the adipocytes would be red stained by Oil Red O, the mineral deposits in osteocytes should be orange-red stained by Alizarin Red S, and the glycosaminoglycans- (GAG-) rich matrix produced by chondrocytes should be red stained by Safranin O.

In order to confirm the multidifferentiation potentials of disk stem cells, the disc fibroblasts (DFCs) were also cultured in the same culture conditions as AFSCs and NPSCs and tested by the same assay used for testing AFSCs and NPSCs.

### 2.5. Quantitative Real-Time RT-PCR (qRT-PCR) for Gene Analysis of Differentiated Cells

Multidifferentiation potential of both AFSCs and NPSCs was further tested by gene expression using qRT-PCR according to the published protocol [14, 15]. After 21-day culture with differentiation media, RNA was extracted from the cells using an RNeasy Mini Kit with an on-column DNase I digest (Qiagen). Total RNA (1 *μ*g) was added to a 20 *μ*L reaction mixture and used for first-strand cDNA synthesis by reverse transcription with SuperScript II kit (Invitrogen). The cDNA was synthesized by two steps, the first step was 65°C for 5 min and cooling for 1 min at 4°C, and the second step was 42°C for 50 min and 72°C for 15 min. The gene expression was tested by qRT-PCR using SYBR Green PCR Kit (Qiagen). Each 2 *μ*L of cDNA sample which contained 100 ng RNA was tested in a 25 *μ*L PCR reaction mixture using a Real-Time PCR System (Step One Plus, AB, Applied Biosystems). The samples were heated for 30 seconds at 95°C for an initial denaturation; then PCR was performed for 50 cycles; each cycle included steps for template denaturation at 95°C for 50 seconds, primer annealing at 56°C for 50 seconds, and primer extension at 72°C for 40 seconds. After a 10-minute final extension at 70°C, the PCR reaction was terminated. The following rabbit specific primers were used for testing gene expression on collagen type I (Col-I), collagen type II (Col-II), collagen type III (Col-III), peroxisome proliferators-activated receptor *γ* (PPAR*γ*), Runx-2, and Sox-9. Glyceraldehyde-3-phosphate dehydrogenase (GAPDH) was used as an internal control.  Table 1 showed the forward and reverse primer sequences designed according to previous publications [8, 17, 18]. All primers were obtained from Invitrogen. The relative expression levels of each gene were obtained from at least three independent experiments.

### 2.6. Statistical Analysis

Data were obtained from at least three independent experiments and expressed as mean ± SD. For statistical data analysis, one-way ANOVA, Fisher's PLSD test, and two-tailed Student's *t*-test were used. When *p* value was less than 0.05, the differences between two groups were considered significant.

## 3. Results

### 3.1. Colony Formation of AFSCs and NPSCs

**Figure 1:**
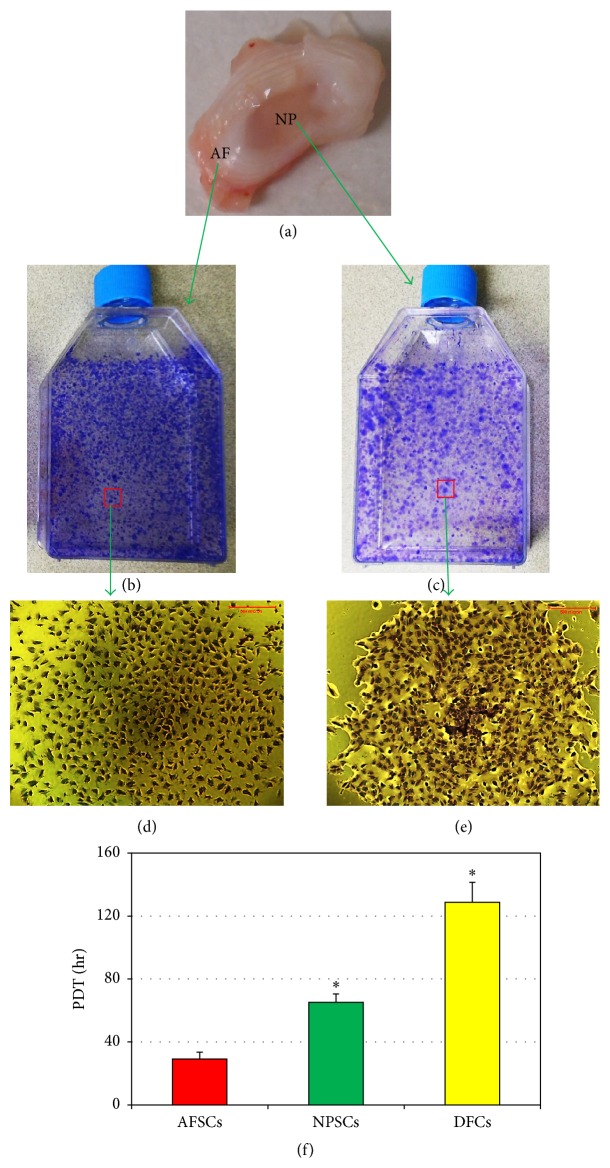
The proliferation and colony formation of disc cells isolated from intervertebral discs of 3-month-old rabbits. (a) The gross appearance and the structure of rabbit intervertebral disc used for cell isolation. (b, c) Colony formation of the cells isolated from AF tissue samples (b) or NP tissue samples (c). (d, e) A typical colony formed by the cells isolated from AF tissue samples (d) or NP tissue samples (e) and stained with methyl violet. (f) Population doubling time of disc cells showed that the stem cells isolated from AF labeled as AFSCs grew much faster than those isolated from NP labeled as NPSCs. In addition, the fibroblasts derived from rabbit discs labeled as DFCs grew slower than both AFSCs and NPSCs. Bars: 500 *μ*m. *∗* indicates statistical significance.

Two kinds of IVD stem cells were isolated from AF and NP tissues of rabbit lumbar spines (Figure 1(a)) and cultured with F-12 medium consisting of 20% FBS and penicillin-streptomycin. The cells appeared in the AF tissue culture plates/flasks on the third day. However, the cells were first found in the NP tissue culture plates/flasks at the fifth day. Both IVD cells attached to the surface of the plate/flask and remained quiescent for one week. The first colony was formed by 309 AFSCs in 7 days of culture; however, the same size colony consisted by 156 NPSCs during 10-day culture. Numerous colonies were then formed of AFSCs after 10 days (Figure 1(b)) and of NPSCs at 17 days (Figure 1(c)). Furthermore, the frequency of colonies formed by AFSCs was 379 ± 34.71 per 500,000 cells, and that by NPSCs was 235 ± 45.23 per 500,000 cells, respectively. The different colony size and density indicated that the proliferation of AFSCs was different from that of NPSCs (Figures 1(b), 1(c), 1(d), and 1(e)). The population doubling time (PDT) demonstrated that AFSCs grew much faster than NPSCs (Figure 1(f)); at passage 1, the PDT for NPSCs was nearly 1.5 times longer than that of AFSCs (Figure 1(f)). However, both disc stem cells, AFSCs and NPSCs, grew faster than disc fibroblasts (Figure 1(f)).

**Figure 2:**
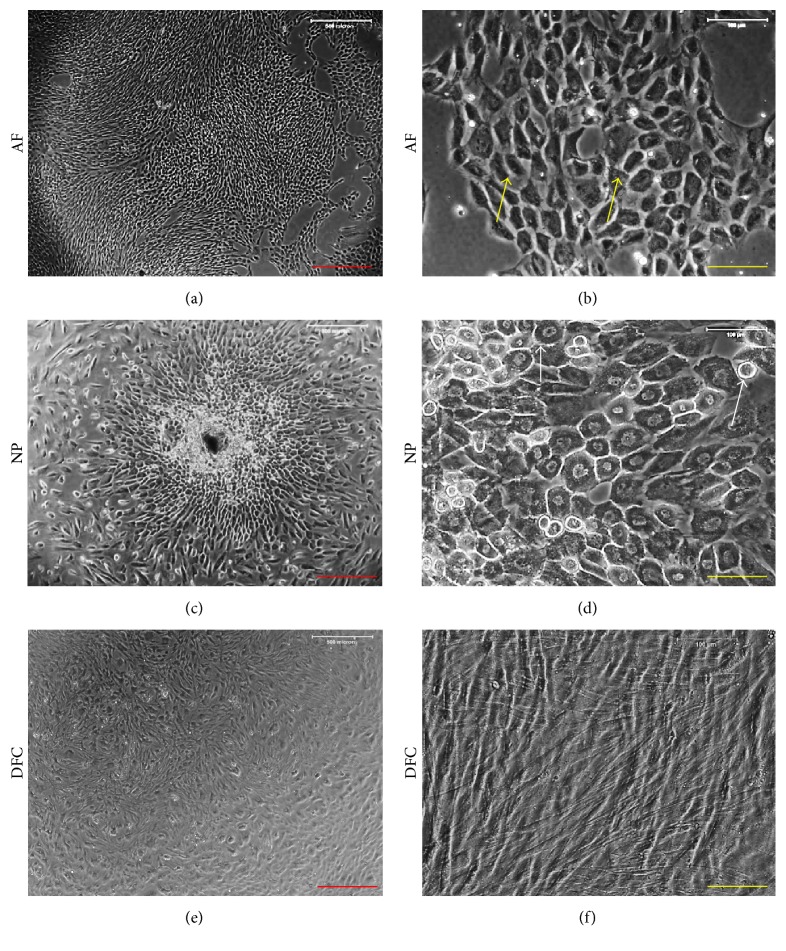
A typical colony of AFSCs (a) and NPSCs (c) and morphology of AFSCs (b), NPSCs (d), and DFCs (e, f) after primary culture for 20 days. The AFSCs showed a cobblestone-like shape ((b), yellow arrows) and NPSCs gave round shape with larger nuclei ((d), white arrows). In contrast, the fibroblasts were highly elongated (e, f). Red Bar: 500 *μ*m. Yellow Bar: 100 *μ*m.

In addition, AFSCs showed small cell size (Figure 2(a)) with a cobblestone-like shape and small nuclei (Figure 2(b)), while NPSCs gave a larger cell size (Figure 2(c)) with a round shape and large nuclei (Figure 2(d)). In contrast, the fibroblasts derived from both AF and NP tissues were highly elongated (Figures 2(e) and 2(f)). The different cell shapes suggested that disc stem cells (AFSCs and NPSCs) were a different type of cells from disc fibroblasts (DFCs).

### 3.2. Stem Cell Marker Expression of AFSCs and NPSCs

**Figure 3:**
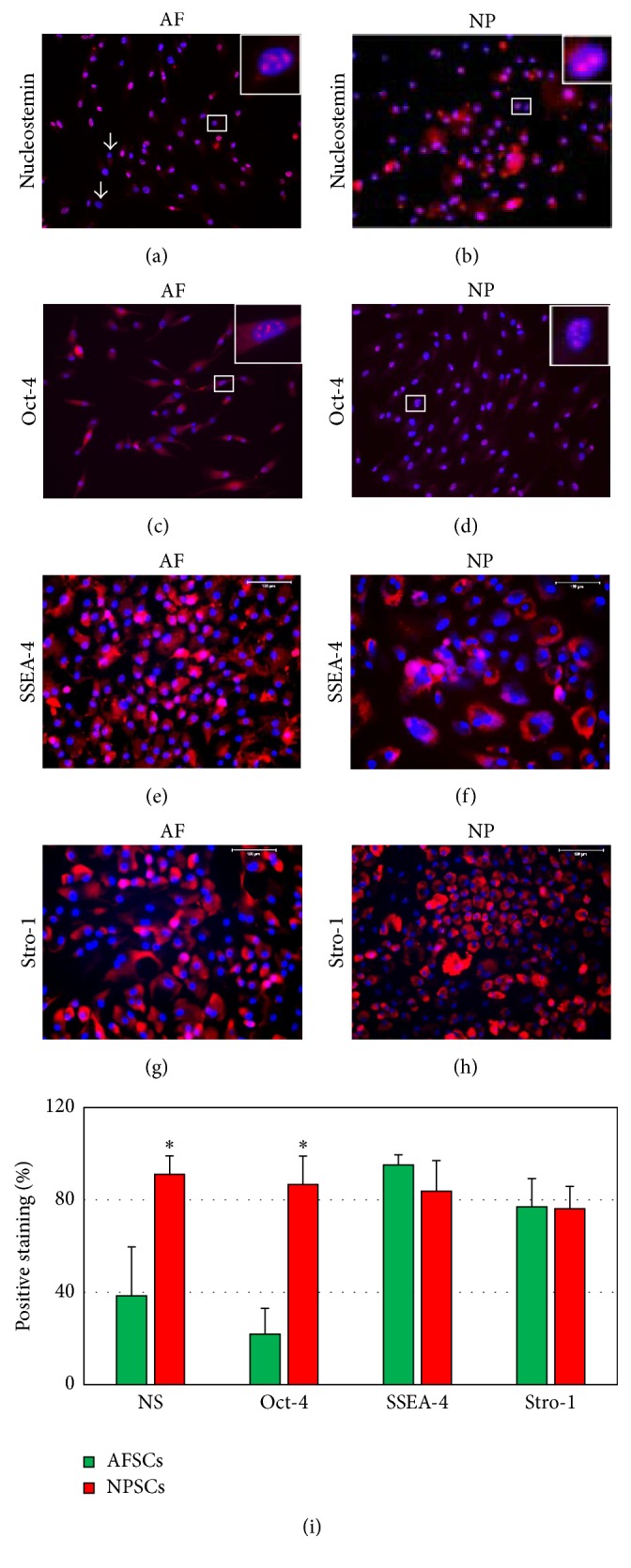
Stem cell marker expression of nucleostemin (NS; (a), (b)), Oct-4 (c, d), SSEA-4 (e, f), and stro-1 (g, h) for AFSCs (a, c, e, and g) and NPSCs (b, d, f, and h) determined by immunocytochemistry (a)–(h) and analyzed by semiquantification (i). Less than 40% of AFSCs expressed nucleostemin (a, i), whereas more than 90% of NPSCs were positively stained indicating nucleostemin (b, i). About 28% of AFSCs presented Oct-4 (c, i) compared to 86% for NPSCs (d, i). There was no significant difference in the expression of SSEA-4 and stro-1 for AFSCs (e, g, i) and NPSCs (f, h, i). Inset images were the enlarged images in the respective square. Bar: 100 *μ*m.

The “stemness” of AFSCs and NPSCs was tested by immunocytochemistry. After 4-day culture at passage 1, both AFSCs and NPSCs were positively stained for nucleostemin (Figures 3(a) and 3(b)), Oct-4 (Figures 3(c) and 3(d)), SSEA-4 (Figures 3(e) and 3(f)), and stro-1 (Figures 3(g) and 3(h)). The semiquantification of results indicated that the “stemness” of AFSCs and NPSCs was different (Figure 3(i)). NPSCs expressed more “stemness” than AFSCs. Less than 40% of AFSCs expressed nucleostemin (Figure 3(a)), whereas more than 90% of NPSCs exhibited nucleostemin (Figure 3(b)). Similarly, about 28% of AFSCs expressed Oct-4 staining (Figure 3(c)), while more than 86% of NPSCs were positively stained by Oct-4 (Figure 3(d)). Both disk stem cells, AFSCs and NPSCs, expressed SSEA-4 and Stro-1 without significant difference (Figures 3(e)–3(h)).

### 3.3. Self-Renewal of AFSCs and NPSCs

**Figure 4:**
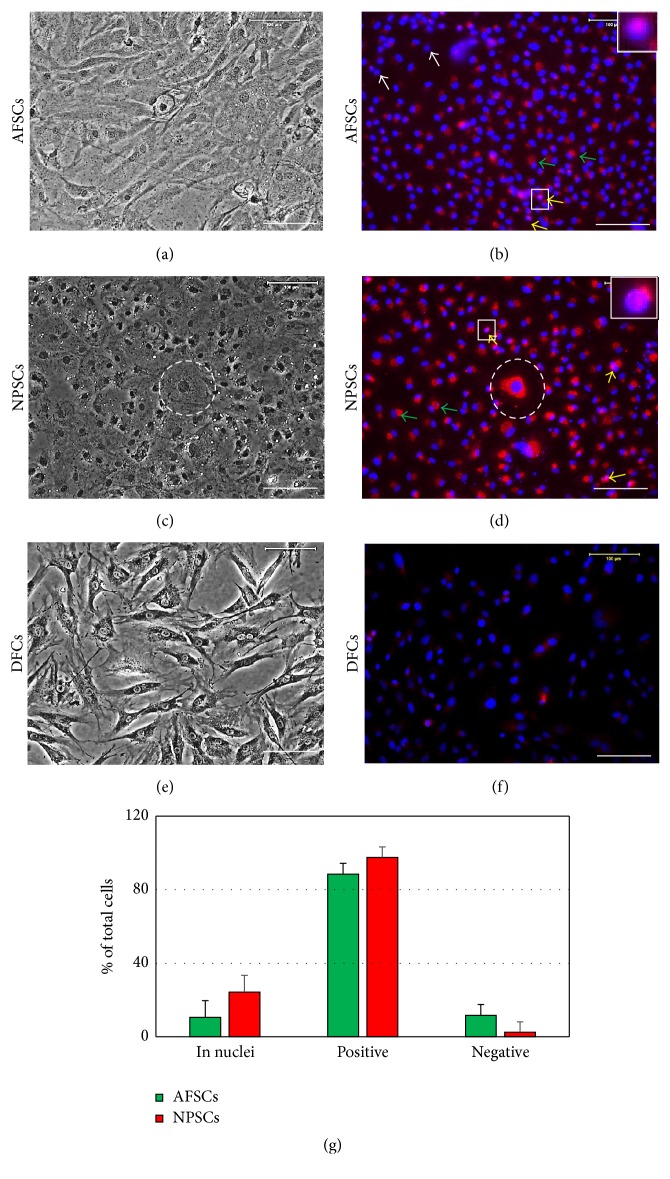
Morphology and nucleostemin expression of AFSCs (a, b) and NPSCs (c, d) at passage 5. Both AFSCs and NPSCs expressed nucleostemin ((b, d), yellow arrows and inset images) at higher passages, whereas the expression levels of nucleostemin decreased (g) and much nucleostemin released from nuclei ((b, c), green arrows). Furthermore, some senescence-like cells were found in NPSCs ((c, d), white circles) and some AFSCs started differentiation and no longer expressed nucleostemin ((b), white arrows). On the other hand, fibroblasts isolated from rabbit discs were negatively stained for nucleostemin (e, f). Semiquantification showed that although more than 80% of both disc stem cells were positively stained indicating nucleostemin at passage 5 (g), about 10% of AFSCs and 23% of NPSCs were stained indicating nucleostemin in cell nuclei (b, d, and g), the other cells released nucleostemin to cytoplasm (b, d, and g), and more than 11% of AFSCs were negatively stained showing nucleostemin (b, g). However, only 2.4% of NPSCs were negatively stained for nucleostemin (d, g). Inset images were the enlarged images in the respective square. Bar: 100 *μ*m.

After repetitive passage, some of AFSCs and NPSCs still expressed stem cell marker nucleostemin (Figures 4(b) and 4(d); inset images). These results indicated that both AFSCs and NPSCs were able to undergo self-renewal.

However, AFSCs became elongated at passage 5 (Figure 4(a)), a typical fibroblast phenotype, and some AFSCs no longer expressed nucleostemin (Figure 4(b), white arrows), suggesting that these AFSCs have differentiated into fibroblasts. In contrast, NPSCs, even after 5 passages, still maintained “stemness” as evidenced by the exhibition of their round shape (Figure 4(c)) and high levels of nucleostemin (Figure 4(d), yellow arrows). However, the level of nucleostemin at passage 5 was lower than that at passage 1 (Figure 3(a)) and positively stained nucleostemin has moved out of nuclei (Figures 4(b) and 4(d), green arrows). More than 24% of NPSCs at passage 5 were still positively stained for nucleostemin in their nuclei (Figures 4(d) and 4(g); yellow arrows); however, less than 11% of AFSCs were stained for nucleostemin in their nuclei (Figures 4(b) and 4(g); yellow arrows). Furthermore, some senescent cell was found in NPSCs after 5 passages (Figure 4(d), white circles). These results indicated that some NPSCs were unactive stem cells and some NPSCs have lost their proliferative ability for self-renewal. In the other hand, the disc fibroblasts had elongated shape (Figure 4(e)) and do not express nucleostemin (Figure 4(f)).

**Figure 5:**
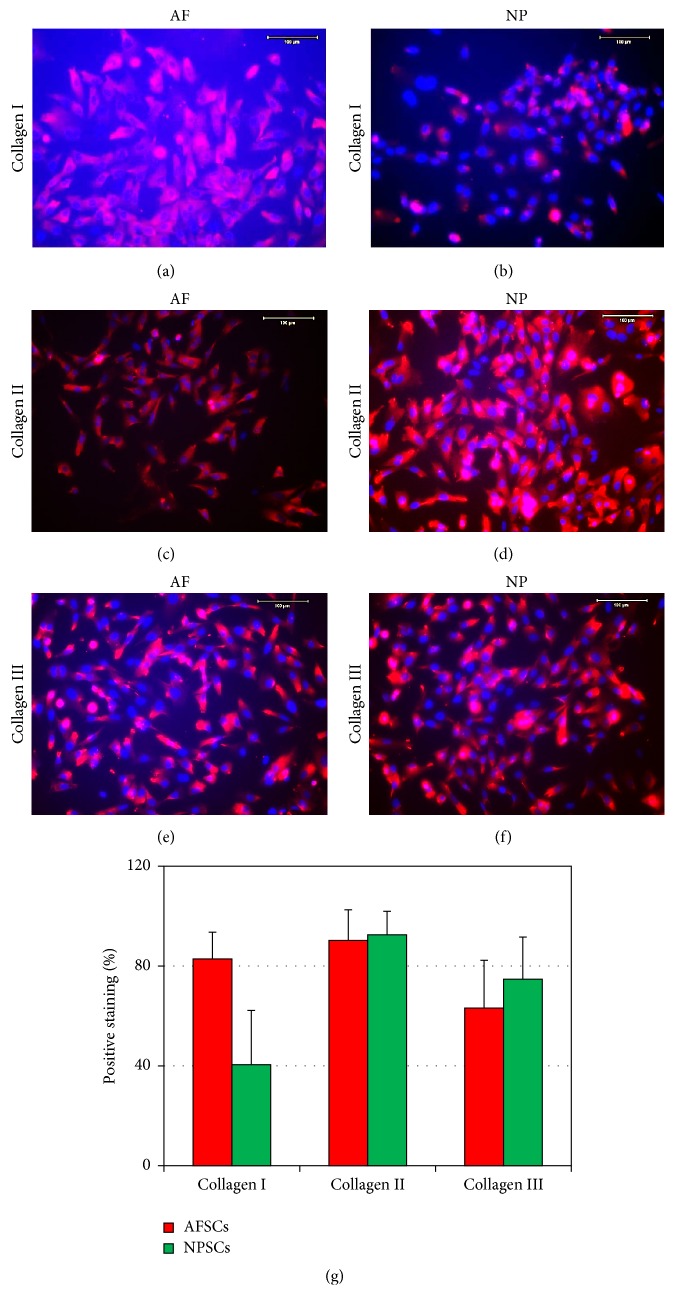
Collagen expression of AFSCs (a, c, and e) and NPSCs (b, d, and f) determined by immunocytochemistry (a)–(f) and analyzed by semiquantification (g). More than 85% of AFSCs expressed collagen type I (a, g), whereas less than 40% of NPSCs were positively stained for collagen type I (b, g). More than 80% of NPSCs and AFSCs expressed collagen type II (c, d, and g). There was no significant difference in the expression of collagen type III for AFSCs (e, g) and NPSCs (f, g). Bars: 100 *μ*m.

Collagen type I and type II are two specific cartilage proteins. Immunostaining showed that more than 85.35% of AFSCs were positively stained for collagen I (Figure 5(a)), whereas only 38.31% of NPSCs were positively stained for collagen I (Figure 5(b)). Moreover, more than 80% of NPSCs and AFSCs expressed collagen type II (Figures 5(c), 5(d), and 5(g)). In addition, collagen type III was presented by both AFSCs and NPSCs without significant difference (Figures 5(e) and 5(f)).

### 3.4. Multidifferentiation Potential of AFSCs and NPSCs

**Figure 6:**
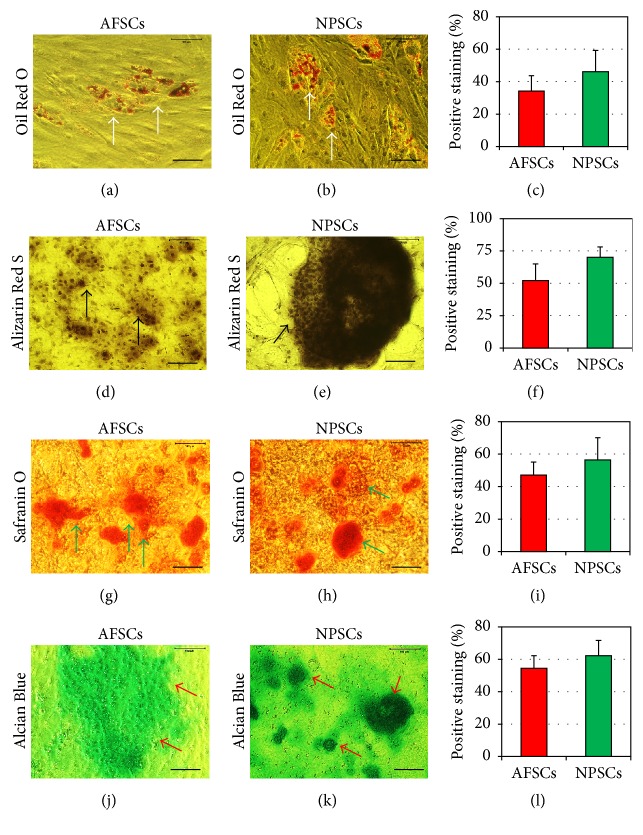
Histochemical staining of differentiated stem cells and semiquantification of the extent of cell differentiation. Both AFSCs and NPSCs were able to differentiate into adipocytes stained in red by Oil Red O ((a), (b), and (c), white arrows), osteocytes stained in dark brown by Alizarin Red S ((d), (e), and (f), black arrows), and chondrocytes stained in red by Safranin O ((g), (h), and (i), green arrows) and stained in green by Alcian Blue ((j), (k), and (l), red arrows), as shown by the accumulation of lipid droplets ((a), (b), red areas), calcium deposits ((d), (e), dark brown areas), and proteoglycans ((g), (h), red areas; (j), (k) green areas) on cell surfaces. However, the extent of AFSC differentiation was less than that of NPSC differentiation, evidenced by smaller positive staining areas for adipogenesis (a, c), osteogenesis (d, f), and chondrogenesis (g, i, j, l) in AFSCs than in NPSCs. Bars: 100 *μ*m.

After culture in adipogenic differentiation medium for 21 days, more than 34.17% of AFSCs and 46.13% of NPSCs differentiated into adipocytes, evidenced by the formation of lipid droplets stained in red by Oil Red O (Figures 6(a), 6(b), and 6(c), white arrows). Similarly, after growth in the osteogenetic medium for 21 days, more than 52.07% of AFSCs and 70.09% of NPSCs were positively stained in dark brown by Alizarin Red S (Figures 6(d), 6(e), and 6(f), black arrows), indicating osteocyte formation. Finally, both AFSCs and NPSCs in chondrogenic medium differentiated into chondrocytes, as glycosaminoglycans- (GAG-) rich matrix was stained in red by Safranin O (Figures 6(g), 6(h), and 6(i), green arrows) and green by Alcian Blue (Figures 6(j), 6(k), and 6(l), red arrows).

**Figure 7:**
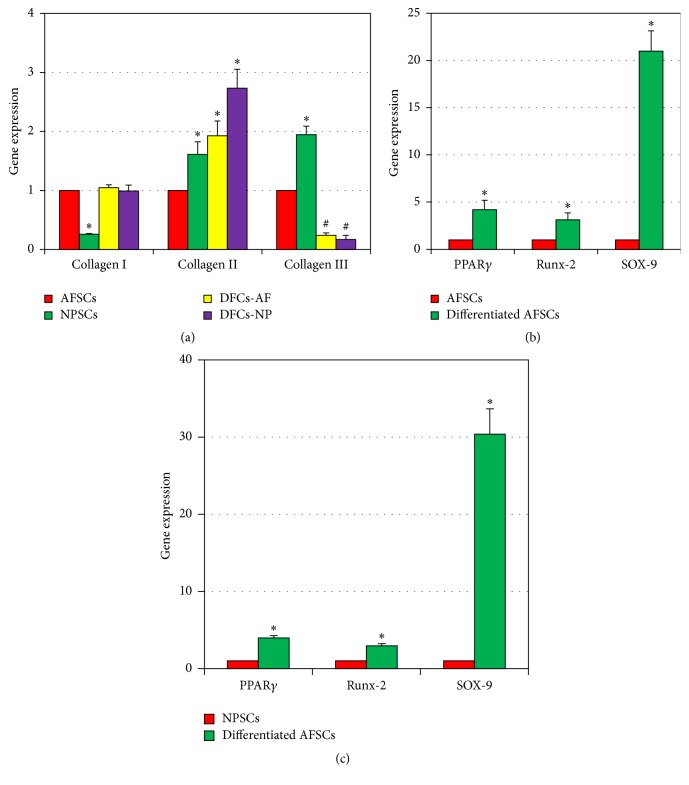
Gene expression of disc stem cells and disc fibroblasts cultured with differentiation media for 21 days. (a) The gene expression of collagen types I, II, and III on disc stem cells (AFSCs and NPSCs) and disc fibroblasts (DFCs-AF and DFCs-NP). NPSCs expressed two times lower collagen type I (^*∗*^ *p* < 0.05 when compared to AFSCs) and higher collagen type III than the other three types of cells, AFSCs, DFCs-AF, and DFCs-NP (^*∗*^ *p* < 0.05 when compared to AFSCs; ^#^ *p* < 0.01 when compared to NPSCs). However, higher collagen type II expression was found in NPSCs, DFCs-AF, and DFCs-NP compared to that in AFSCs (^*∗*^ *p* < 0.05 when compared to AFSCs). (b) Multidifferentiation potential of AFSCs cultured with various differentiation media for 21 days. The gene expression in differentiated AFSCs was found to be 4 times higher on PPAR*γ* (adipocyte marker gene), 2 times higher on Runx-2 (osteocyte marker gene), and 20 times higher on SOX-9 (chondrocyte marker gene) compared to those in AFSCs. AFSCs: the AFSCs were cultured with normal growth medium (DMEM-10% FBS) for 21 days; differentiated AFSCs: the AFSCs were cultured with either adipogenesis or osteogenesis or chondrogenesis media for 21 days (^*∗*^ *p* < 0.05 when compared to AFSCs cultured with normal growth medium). (c) Multidifferentiation potential of NPSCs cultured with various differentiation media. The gene expression in differentiated NPSCs was found to be 3 times higher on PPAR*γ* (adipocyte marker gene), 2 times higher on Runx-2 (osteocyte marker gene), and 29 times higher on SOX-9 (chondrocyte marker gene) compared to those in NPSCs. NPSCs: the NPSCs were cultured with normal growth medium (DMEM-10% FBS) for 21 days; differentiated NPSCs: the NPSCs were cultured with either adipogenesis or osteogenesis or chondrogenesis media for 21 days (^*∗*^ *p* < 0.05 when compared to NPSCs cultured with normal growth medium).

Furthermore, the gene expression of collagen types I, II, and III on disc stem cells (AFSCs and NPSCs) and disc fibroblasts (DFCs-AF and DFCs-NP) was tested by qRT-PCR. Both AFSCs and DFCs-AF expressed high-level collagen type I (Figure 7(a)). However, NPSCs expressed lower concentration of the collagen type I than the other three types of cells, AFSCs, DFCs-AF, and DFCs-NP (Figure 7(a), ^*∗*^ *p* < 0.05 when compared to AFSCs). All cells expressed higher concentration of collagen type II (Figure 7(a)). However, a lower concentration of collagen type III was found in both disc fibroblasts (Figure 7(a), ^#^ *p* < 0.05 when compared to AFSCs). Multidifferentiation potential of AFSCs and NPSCs cultured with/without adipogenic induction medium, osteogenic induction medium, and chondrogenic induction medium was also tested by qRT-PCR (Figures 7(b) and 7(c)). The gene expressions of PPAR*γ* (an adipocyte marker), Runx-2 (an osteocyte marker), and SOX-9 (a chondrocyte marker) were upregulated in both AFSCs and NPSCs cultured with adipogenesis, osteogenesis, and chondrogenesis media, respectively (Figure 7(b), ^*∗*^ *p* < 0.05, when compared to AFSCs, cultured with normal growth medium; Figure 7(c), ^*∗*^ *p* < 0.05, when compared to NPSCs, cultured with normal growth medium).

## 4. Discussion

The aims of this study were to determine whether adult stem cells exist in intervertebral discs of rabbits and whether the stem cells isolated from nucleus pulposus (NP) and annulus fibrosus (AF) have common properties in their phenotypes. For these aims, AFSCs and NPSCs were isolated from NP and AF regions, two typical tissues of intervertebral discs of rabbits, and their “stemness” including differentiation potential, cell marker expression, morphology, and proliferative potential were investigated. The results showed that these AFSCs and NPSCs have characteristics of stem cells, such as clonogenicity, self-renewal, and multipotency. Moreover, both populations expressed stem cell markers nucleostemin, Oct-4, SSEA-4, and Stro-1, as well as IVD cell markers, including collagen type I, collagen type II, and collagen type III. However, a smaller proportion of AFSCs expressed Oct-4 and Stro-1 compared to NPSCs. NPSCs also grew slower and formed smaller and fewer colonies than AFSCs. Finally, AFSCs lost their “stemness” faster than NPSCs. However, more quiescent or senescent cells were found in NPSCs at the passages higher than 5.

It is well known that nucleus pulposus (NP) and annulus fibrosus (AF) are two important components in intervertebral discs. NP cells are chondrocyte-like cells situated within a collagen type II and proteoglycan-enriched matrix that absorbs the spinal load. AF cells are fibroblast-like cells embedded in a collagen type I enriched expansion-resistant matrix. We found that fibroblasts isolated from both NP and AF tissues essentially lacked transdifferentiation potential; moreover, these disc fibroblasts did not express stem cell markers including nucleostemin, Oct-4, SSEA-4, and Stro-1. Morphologically, AFSCs and NPSCs in culture differ from fibroblasts in that the stem cells exhibited a cobblestone shape or round shape, whereas the disc fibroblasts spread out and were highly elongated, a characteristic shape of fibroblasts in confluent conditions. Finally, both stem cells isolated from AF and NP proliferated significantly faster than disc fibroblasts.

The findings that both stem cells isolated from AF and NP were capable of differentiating into adipocytes, osteocytes, and chondrocytes suggest that both stem cells, AFSCs and NPSCs, may play a key role in intervertebral disc degenerative diseases. The fibroblasts did not exhibit this capability of differentiating.

Despite the high prevalence of intervertebral disc disease, little is known about changes in intervertebral disc cells and their regenerative potential with intervertebral disc degeneration [19]. The intervertebral disc undergoes degenerative changes earlier in life than do other tissues of major organ systems that are comprised of long-lived cells that are normally replaced infrequently [20, 21]. In degenerative disc disease, there is an increase in cell proliferation and formation of cell clusters as well as an increase in cell death. The cartilage end plate undergoes thinning, altered cell density, the formation of fissures, and sclerosis of the subchondral bone [21]. Therefore, by virtue of their ability to differentiate into adipocytes, osteocytes, and chondrocytes, AFSCs and NPSCs could be responsible for the production of abnormal matrix components including fatty tissue formation, glycosaminoglycan degeneration, and calcifications seen in the degenerative intervertebral disc.

In the present study, we found that adult stem cells, AFSCs and NPSCs, exist in the intervertebral disc of rabbits. Both AFSCs and NPSCs expressed stem cell markers, nucleostemin, Oct-4, SSEA-4, and Stro-1. Oct-4 is a transcription factor that is typically expressed in embryonic stem cells during development and is essential for establishing and maintaining undifferentiated pluripotent stem cells [22]. Like previous studies which showed that Oct-4 was expressed in human and mouse BMSCs [23], we also found that AFSCs and NPSCs expressed Oct-4, encouraging future examination of whether the multipotency of AFSCs and NPSCs demonstrated in this study depends on Oct-4 expression.

It is known that SSEA-4 is developmentally regulated during early embryogenesis and is widely used as a marker to monitor the differentiation of both mouse and human embryonic stem cells [24]. Therefore, we used SSEA-4 as one of the markers for AFSCs and NPSCs. We found that both AFSCs and NPSCs expressed SSEA-4 strongly.

Nucleostemin is only expressed in the nucleoli of stem cells and cancer cells but not in those of committed and terminally differentiated cells [25]. High level of nucleostemin expression found in both AFSCs and NPSCs of rabbits indicated that stem cells were an actively proliferating, self-renewing population of cells in the culture conditions. In contrast, the lack of expression of nucleostemin in disc fibroblasts suggested that disc fibroblasts are terminally differentiated cells without further differentiation potential, as evidenced by current results.

The present results showed that both AFSCs and NPSCs isolated from two important regions of rabbit intervertebral discs were heterogeneous in clonogenicity, multidifferentiation potential, and self-renewal due to the fact that cell populations were a mixture of stem cells and progenitor cells. We found that these different cell populations did not undergo complete differentiation into adipocytes, osteocytes, and chondrocytes when cultured in respective differentiation media. Furthermore, not all individual cells expressed stem cell markers. Finally, we noted that AFSCs lost “stemness” faster than NPSCs, and NPSCs grew slower than AFSCs and remained quiescent at higher passages.

The diseases of the intervertebral disc, degenerative disc disease and scoliosis, are both characterized by changes in the extracellular matrix components [26]. Degeneration of the intervertebral disc has been implicated in chronic low back pain [9]. Collagens are the main extracellular matrix components of discs. Normal NP consists of fibers and proteoglycans, which form a hydrophilic molecular complex that generates a swelling pressure sufficient to separate adjacent vertebrae [27]. As the disc ages, the degeneration occurs, osmotic pressure is lost in the nucleus, dehydration occurs, and the disc loses its height [28]. Our results showed that AFSCs produced a high concentration of collagen type I and NPSCs expressed high concentration of collagen type II at lower passages. However, at higher passages, both types of disc stem cells started losing their “stemness,” AFSCs started their differentiation, and NPSCs changed to senescence. Our findings may explain above-mentioned clinical problems which may be caused by stem cell proliferation and wrong differentiation. An important finding of this study is that both AFSCs and NPSCs expressed strongly collagen type III. It has been reported that collagen type III presented in AF and NP regions during human and rat IVD development has a role in IVD development [29, 30]. However, increased collagen type III has also been reported paracellularly in degenerating discs so that it may also be present during attempted repair or other degenerative processes [7].

The intervertebral disc is commonly considered to contain only chondrocytes or disc fibroblasts. Our findings suggested that rabbit intervertebral discs contain stem cells. While this study shows that disc stem cells from both AF and NP exhibit similar patterns in directed differentiation and gene expression, they display significant differences in colony formation and cell proliferation rate. The reasons for these differences are not clear but may reflect inherent differences between the two tissues of the intervertebral disc* in vivo*. Further studies will look into the properties of disc stem cells at the individual cell level rather than at the population level. In addition, further research should investigate how to enhance the proliferation and maintain the “stemness” of AFSCs and NPSCs* in vitro *and* in vivo*. Finally, further study should determine whether AFSCs and NPSCs can be used for more effective repair and regeneration of degenerative intervertebral discs.

## Figures and Tables

**Table 1:** Primers for qRT-PCT analysis.

Gene	Size (bp)	Primers	Type
Collagen I	81	5′-CTG ACT GGA AGA GCG GAG AGT AC-3′	Forward
		5′-CCA TGT CGC AGA AGA CCT TGA-3′	Reverse

Collagen II	84	5′-TGG GTG TTC TAT TTA TTT ATT GTC TTC CT-3′	Forward
		5′-GCG TTG GAC TCA CAC CAG TTA GT-3′	Reverse

Collagen III	255	5′-TTATAAACCAACCTCTTCCT-3′	Forward
		5′-TATTATAGCACCATTGAGAC-3′	Reverse

PPAR*γ*	200	5′-TGG GGA TGT CTC ATA ATG CCA-3′	Forward
		5′-TTC CTG TCA AGA TCG CCC TCG-3′	Reverse

Sox-9	79	5′-AGT ACC CGC ACC TGC ACA AC-3′	Forward
		5′-CGC TTC TCG CTC TCG TTC AG-3′	Reverse

Runx-2	70	5′-TGA TGA CAC TGC CAC CTC TGA-3′	Forward
		5′-GCA CCT GCC TGG CTC TTC T-3′	Reverse

GAPDH	107	5′-ACT TTG TGA AGC TCA TTT CCT GGT A-3′	Forward
		5′-GTG GTT TGA GGG CTC TTA CTC CTT-3′	Reverse
